# A role for DNA-dependent activator of interferon regulatory factor in the recognition of herpes simplex virus type 1 by glial cells

**DOI:** 10.1186/1742-2094-8-99

**Published:** 2011-08-12

**Authors:** Samantha R Furr, Vinita S Chauhan, Megan J Moerdyk-Schauwecker, Ian Marriott

**Affiliations:** 1 Department of Biology, University of North Carolina at Charlotte, Charlotte, NC 28223, USA

**Keywords:** DNA-dependent activator of IFN regulatory factor, microglia, astrocytes, neuroinflammation, innate immunity, encephalitis, herpes simplex virus-1

## Abstract

**Background:**

The rapid onset of potentially lethal neuroinflammation is a defining feature of viral encephalitis. Microglia and astrocytes are likely to play a significant role in viral encephalitis pathophysiology as they are ideally positioned to respond to invading central nervous system (CNS) pathogens by producing key inflammatory mediators. Recently, DNA-dependent activator of IFN regulatory factor (DAI) has been reported to function as an intracellular sensor for DNA viruses. To date, the expression and functional role of DAI in the inflammatory responses of resident CNS cells to neurotropic DNA viruses has not been reported.

**Methods:**

Expression of DAI and its downstream effector molecules was determined in C57BL/6-derived microglia and astrocytes, either at rest or following exposure to herpes simplex virus type 1 (HSV-1) and/or murine gammaherpesvirus-68 (MHV-68), by immunoblot analysis. In addition, such expression was studied in ex vivo microglia/macrophages and astrocytes from uninfected animals or mice infected with HSV-1. Inflammatory cytokine production by glial cultures following transfection with a DAI specific ligand (B-DNA), or following HSV-1 challenge in the absence or presence of siRNA directed against DAI, was assessed by specific capture ELISA. The production of soluble neurotoxic mediators by HSV-1 infected glia following DAI knockdown was assessed by analysis of the susceptibility of neuron-like cells to conditioned glial media.

**Results:**

We show that isolated microglia and astrocytes constitutively express DAI and its effector molecules, and show that such expression is upregulated following DNA virus challenge. We demonstrate that these resident CNS cells express DAI *in situ*, and show that its expression is similarly elevated in a murine model of HSV-1 encephalitis. Importantly, we show B-DNA transfection can elicit inflammatory cytokine production by isolated glial cells and DAI knockdown can significantly reduce microglial and astrocyte responses to HSV-1. Finally, we demonstrate that HSV-1 challenged microglia and astrocytes release neurotoxic mediators and show that such production is significantly attenuated following DAI knockdown.

**Conclusions:**

The functional expression of DAI by microglia and astrocytes may represent an important innate immune mechanism underlying the rapid and potentially lethal inflammation associated with neurotropic DNA virus infection.

## Background

The neurotropic DNA virus herpes simplex virus type 1 (HSV-1) is capable of causing severe necrotizing encephalitis and accounts for 95% of all fatal cases of sporadic viral encephalitis [1]. Untreated HSV-1 encephalitis has a 70% mortality rate and patients who receive early treatment have only a 40% chance of recovery without significant neurological deficits. Furthermore, the overall mortality rate of HSV-1 encephalitis in the US remains at 30% despite improvements in diagnosis and therapy [2]. It has recently been shown that HSV-1-mediated cytokine and chemokine production contributes to CNS damage following *in vivo *infection suggesting that an overzealous host response is a major contributor to the neuropathology associated with acute viral encephalitis [3-7]. However, the mechanisms underlying the onset of such damaging neuroinflammation have not been defined.

The rapid onset of inflammation following CNS infection suggests that resident glial cells play a pivotal role in the initiation and progression of encephalitis. Microglia and astrocytes are resident cells of the CNS cells and are susceptible to HSV-1 infection [8]. Both of these cell types are now recognized to have innate immune functions and respond to invading pathogens by producing soluble mediators that can promote inflammation and leukocyte recruitment across the blood-brain barrier [9-11]. Importantly, microglia have been shown to produce significant levels of the proinflammatory cytokines TNF-α and IL-6 in response to HSV-1 infection [12]. While host immune cells have been shown to recognize HSV-1 or HSV-2 via cell-surface/endosomal pattern recognition receptors including Toll-like receptor (TLR) 2, and TLR9 [13,14], the means by which resident CNS cells perceive DNA virus infection and initiate inflammatory cytokine production have not been defined.

Recently, the cytosolic protein, DNA-dependent activator of interferon-regulatory factors (DAI; also known as Z-DNA-binding protein 1 (ZBP1)), has been reported to function as an innate sensor of intracellular viral DNA [15-18]. This molecule has been shown to recognize double-stranded DNA in its canonical B helical form (B-DNA) [17,18] and elicit type-I IFN production in a TANK-binding kinase 1 and interferon regulatory factor 3 dependent, but TLR9-independent, manner [16,19]. Importantly, this cytosolic sensor has been reported to mediate type I interferon and inflammatory cytokine production by HSV-1-infected murine fibroblasts [17]. To date, DAI expression has not been reported in microglia or astrocytes, and a role for this putative viral sensor in the innate immune responses of resident CNS cells to viral challenge has not been explored. In the present study, we provide the first demonstration of constitutive and inducible expression of DAI and its downstream effector molecules by glial cells both *in vitro *and *in situ*. Importantly, we confirm the functional status of DAI in primary microglia and astrocytes and demonstrate that this viral sensor plays a significant role in the inflammatory responses of resident CNS cells to HSV-1 challenge.

## Materials and methods

### Isolation of primary murine microglia and astrocytes

Murine neonatal brain microglia and astrocytes were isolated as described previously [20-22]. Microglia were cultured in RPMI 1640 with 10% FBS and 20% conditioned medium from LADMAC cells (ATCC, CRL-2420) as a source of colony stimulating factor-1 while astrocytes were cultured in RPMI 1640 containing 10% FBS. Cells isolated in this manner were >95% and >97% authentic microglia and astrocytes, respectively, as assessed by their characteristic morphology and by expression of the microglial/macrophage markers CD11b and F4/80 or the astrocyte marker glial fibrillary acidic protein (GFAP) as determined by immunofluorescent microscopy.

### Preparation of viral stocks

HSV-1 viral stocks were prepared by infecting monolayer cultures of Vero cells (ATCC; CCL-81) with HSV-1 (MacIntyre strain from a patient with encephalitis; ATCC, VR-539) at a low multiplicity of infection (MOI) of 0.05 plaque-forming units (PFU) per cell and incubated for 48 hours at 34°C in SFM4MegaVir protein-free medium (Thermo Scientific Hyclone, Waltham, MA). Cells were removed with trypsin and pulse sonicated (Vibra Cell; Sonics & Materials Inc., Newtown, CT) to release intact virions. The sonicated material was centrifuged to remove unwanted cellular debris and the viral titers in the cell-free supernatant was quantified using a standard plaque assay of serial dilutions of HSV-1 on Vero cells at 34°C. MHV-68 viral stocks were prepared by infecting monolayer cultures of BHK-21 cells (ATCC, CCL-10) at a low viral MOI of 0.1 PFU per cell. After 24 hour, the cells were removed with trypsin and pulse sonicated to release intact virions. The sonicated material was centrifuged to remove unwanted cellular debris and the supernatant containing virus was aliquoted and stored at -80°C. The medium containing released virus was collected and viral titers were quantified using a standard plaque assay of threefold serial dilutions on NIH-3T3 cells at 34°C.

### *In vitro *infection with HSV-1 and MHV-68

Isolated primary microglia and astrocytes were infected with HSV-1 or MHV-68 at MOIs of 0.01, 0.1, 1, and 10 PFU per cell and the viruses were allowed to adsorb for 1 hour prior to washing to remove nonadherent viral particles. Cultures were maintained for 12 or 24 hours prior to collection of culture supernatants or preparation of whole cell protein isolates.

### Western blot analyses for DAI, RIP3, STING, and a HSV-1 gene product

Western blot analyses for the presence of DAI, receptor-interacting protein 3 (RIP3), stimulator of interferon genes (STING), and the HSV-1 gene product glycoprotein G-1 (gG1) in microglia, astrocytes, and whole brain samples were performed as described previously by our laboratory [21,23,24]. After incubation with a rabbit polyclonal antibody against DAI (Abcam, Cambridge, MA), RIP3 (Abcam, Cambridge, MA), STING (Abcam, Cambridge, MA) or a mouse monoclonal antibody against gG1 (Abnova, Taipei, Taiwan) for 24 hours at 4°C, blots were washed and incubated in the presence of an horseradish peroxidase (HRP)-conjugated anti-rabbit antibody (Cell Signaling, Danvers, MA) or an HRP-conjugated anti-mouse antibody (Cell Signaling, Danvers, MA), respectively. Bound enzyme was detected with the Super Signal system (Thermo Scientific, Rockford, IL). The expression level of an unidentified non-specific protein present in Coomassie blue stained membranes was determined to confirm equal protein loading in each lane (loading control: lc). Immunoblots shown are representative of at least three separate experiments.

### Densitometric analyses

Densitometric analysis of each immunoblot was performed using ImageJ obtained from the NIH Web site http://rsbweb.nih.gov/ij/download.html. Results are presented as fold increases in the number of arbitrary densitometric units, corrected for background intensity and normalized to the expression of the loading control protein, over those in unstimulated cells.

### *In vivo *HSV-1 infection

HSV-1 (MacIntyre strain) was administered to 4-6 week-old female C57BL/6 mice (Jackson Laboratories) via intranasal (i.n.) infection essentially as described by our laboratory with other viral pathogens [23,25]. Anesthetized animals were untreated or received i.n. HSV-1 administration (1 × 10^5 ^-1 × 10^6 ^PFU) in PBS (final volume of 20 μl). Animals were euthanized at 4 days post-infection and protein isolates were prepared from whole brain tissue homogenates or glial cells isolated from infected and uninfected animals by flow cytometry as described below. All studies were performed in accordance with relevant federal guidelines and institutional policies regarding the use of animals for research purposes.

### Isolation and cytometric analysis of ex vivo CNS cells

Mixed CNS cells were isolated from infected and uninfected animals using a protocol modified from Campanella and coworkers [26] as previously described [27]. Whole brains were rapidly removed and mechanically disrupted in a glass homogenizer, washed, and resuspended in PBS/30% Percoll (Fluka, Sigma Aldrich, St. Louis, MO) solution. This was overlaid on a gradient containing 37% and 70% Percoll solutions and centrifuged at 600 × G for 20 minutes at room temperature. Glial cells were then collected from the interface and washed with PBS. Microglia/macrophages and astrocytes were isolated from the mixed glial preparation by flow cytometry using an R-phycoerythrin conjugated monoclonal antibody directed against mouse CD11b (BD Biosciences, clone M1-70) and an AlexaFluor488-conjugated monoclonal antibody directed against mouse GFAP (Invitrogen, Eugene, OR, clone 131-17719), respectively. Protein isolates were prepared and analyzed for the presence of DAI, STING, RIP3, and viral gG1 by immunoblot analysis.

### *In vitro *stimulation of microglia and astrocytes with the DAI ligand, B-DNA

Poly(dA:dT) double-stranded B-DNA (InvivoGen, San Diego, CA) was directly introduced into microglia and astrocytes at concentrations of 3 μg/ml and 6 μg/ml using FuGENE HD Transfection Reagent (Promega, Madison, WI) according to the manufacturer's instructions. At 6 and 12 hours following transfection, culture supernatants and whole cell protein isolates were collected for analysis. For comparison purposes, cells were exposed to transfection reagent alone or HSV-1 (MOI of 1 and 10) for 24 hours.

### Quantification of IL-6 and TNF-α secretion in glial cell culture supernatants

Specific capture ELISAs were performed to quantify IL-6 and TNF-α as described previously by our laboratory [20-24]. A commercially available ELISA kit was used to measure TNF-α (R&D Systems, Minneapolis, MN) secretion while IL-6 secretion was measured using a rat anti-mouse IL-6 capture antibody (Clone MP5-20F3) and a biotinylated rat anti-mouse IL-6 detection antibody (Clone MP5-C2311) (BD Pharmingen, San Diego, CA).

### siRNA-mediated DAI knockdown

**Figure 1:**
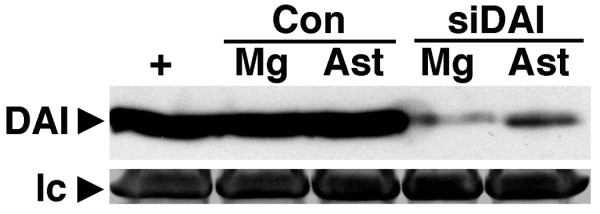
**Efficiency of DAI knockdown in primary murine microglia and astrocytes by small-interfering RNA**. Microglia (Mg) and astrocytes (Ast) were untreated (Con) or transfected with a combination of three different siRNA duplexes targeting murine DAI using FuGENE HD transfection reagent. At 72 hours post-transfection, untreated and transfected cells were exposed to HSV-1 (MOI of 10) for 24 hours and DAI knockdown was confirmed in whole cell lysates by immunoblot analysis. Expression of a non-specific protein band observed following Coomassie blue staining is shown as a loading control (lc). For comparison purposes, DAI expression in murine small intestine tissue is shown (+).

Three validated Stealth RNAi™ small interfering (si)RNA duplexes targeting murine DAI, in addition to a universal negative control siRNA that was not homologous to anything in the vertebrate transcriptome, were purchased from Invitrogen (Carlsbad, CA). Microglia and astrocytes were transfected with a combination of the siRNA duplexes targeting DAI or the negative control siRNA using FuGENE HD transfection reagent as previously described by our laboratory [24]. Antibiotic-free media was replaced with complete media at 6 hours following transfection. Maximal DAI knockdown was achieved in both cell types at 72 hours post-transfection and our ability to markedly reduce DAI expression following HSV-1 infected glial cells using this protocol was confirmed in whole cell lysates by immunoblot analysis (Figure 1). This knockdown protocol was also employed in some studies prior to HSV-1 infection (MOI of 0.01, 0.1, 1, and 10).

### Assessment of soluble neurotoxic mediator production by infected microglia and astrocytes

Transfected or untransfected microglia and astrocytes were uninfected or infected with HSV-1 (MOI of 1 PFU per cell) for 1 hour prior to washing to remove non-adherent viral particles. At 24 hours following infection, the conditioned medium was collected and filtered using a 0.1-μm syringe filter (Sterlitech, Kent, WA) to remove residual HSV-1 particles (180 - 200 nm in diameter) prior to addition to resting CATH.a murine neuron-like cell cultures (ATCC #CRL-11179). Our ability to remove all infectious viral particles from the conditioned medium was verified in parallel experiments by demonstrating the exclusion of the smaller vesicular stomatitis virus (0.1-0.4 nm in length) engineered to express green fluorescent protein as described previously [23,24]. At 4, 8, and 12 hours following filtered conditioned medium addition, the numbers of adherent CATH.a cells were counted in ten microscopy fields and viability was assessed by trypan blue exclusion.

### Statistical analyses

Results of the present studies were tested statistically by one-way ANOVA and Tukey's post hoc test using commercially available software (SAS Institute, Cary, NC). Results were considered statistically significant when a p value of less than 0.05 was obtained.

## Results

### Primary glial cells express DAI and its associated downstream effector molecules

**Figure 2:**
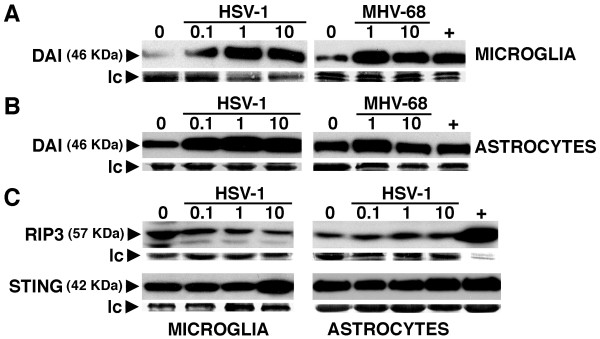
**Cultured primary murine astrocytes and microglia constitutively express the cytoplasmic viral sensor DAI and associated effector molecules, and its expression is elevated following viral challenge**. Cultured microglia (Panels A and C) or astrocytes (Panels B and C) were untreated (0) or infected with HSV-1 (MOI of 0.1, 1 and 10) or MHV-68 (MOI of 1 and 10). At 24 hours post-infection expression of DAI (Panels A and B), RIP3 and STING (Panel C) were determined in whole cell lysates by immunoblot analysis. Expression of a non-specific protein band observed following Coomassie blue staining is shown as a loading control (lc) for each blot. For comparison purposes, DAI expression in murine small intestine tissue is shown (+). Representative results are shown for one of three separate experiments.

To determine whether resident CNS cells express DAI we have assessed the expression of this cytoplasmic viral sensor in isolated primary murine microglia and astrocyte cultures. As shown in Figure 2, both microglia and astrocytes constitutively express detectable levels of DAI as determined by immunoblot analysis although it is noteworthy that resting astrocytes demonstrated higher expression levels than that seen in an equal number of microglia. HSV-1 elicited an upregulation in microglial DAI expression by up to 23.8 fold over that seen in resting cells (Figure 2A). Despite robust constitutive expression, HSV-1 exposure was also able to further increase DAI expression in astrocytes with a maximal increase of 2.2 fold over that seen in unstimulated cells (Figure 2B). Interestingly, the ability of viral challenge to augment DAI expression by primary glial cells was not limited to this neurotropic alphaherpesvirus as the lymphotropic [28] gammaherpesvirus, MHV-68, was also capable of eliciting robust increases in microglial DAI expression (Figure 2A) and causing modest increases in the expression of this molecule in astrocytes (7.3-fold) (Figure 2B).

Finally, to begin to determine whether DAI is functional in glial cells we have investigated whether these cells express RIP3 and STING, two critical downstream effector molecules in DAI signaling [29-31]. As shown in Figure 2C, both microglia and astrocytes constitutively express robust levels of RIP3 and STING. In contrast to DAI expression, STING levels were essentially unaltered following viral challenge (Figure 2C) and while RIP3 expression tended to decrease in microglia following HSV-1 challenge (maximal decrease of 1.5 +/- 0.3 fold; n = 3) and increase in astrocytes (maximal increase of 1.4 +/- 0.2 fold, n = 3) such effects were not statistically significant.

### *In vivo *HSV-1 infection induces DAI expression by microglia/macrophages and astrocytes

**Figure 3:**
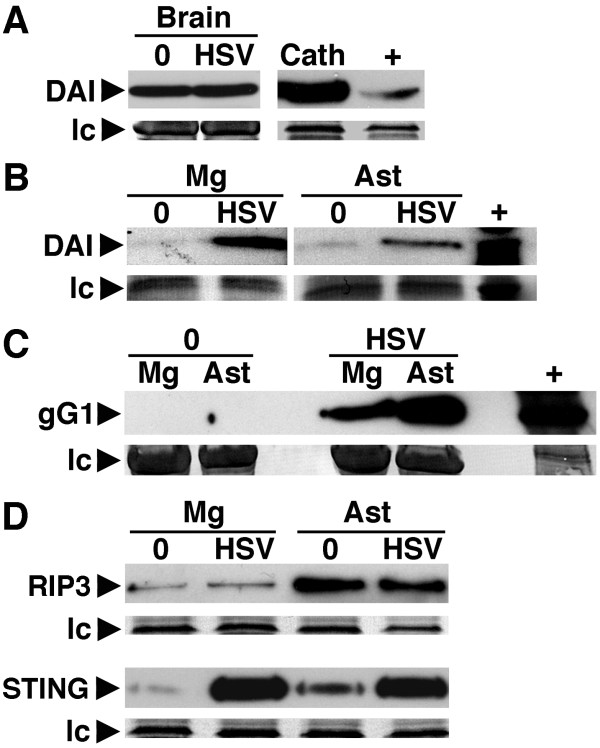
***Ex vivo *glial cells express DAI constitutively and/or inducibly following *in vivo *HSV-1 infection**. Mice were sham-infected (0) or infected with HSV-1 (HSV: 1 × 10^5 ^PFU, i.n.). At 4 days post-infection protein isolates were prepared from whole brain tissue (Brain), or microglia (Mg) or astrocytes (Ast) acutely isolated by flow cytometry, and analyzed for the presence of DAI (Panels A and B), viral gG1 (Panel C), RIP3 and STING (Panel D) by immunoblot analysis. For comparison purposes, DAI expression in the murine neuron-like cell line CATH.a (Panel A: Cath) and murine small intestine tissue (Panels A and B: +), or viral gG1 expression in HSV-1 infected Vero cells (Panel C: +) is shown. Expression of a non-specific protein band observed following Coomassie blue staining is shown as a loading control (lc) for each blot. Immunoblots shown are representative of three separate experiments.

To determine if glial cells express DAI *in situ*, we first assessed the expression of this viral sensor in ex vivo microglia/macrophages and astrocytes isolated from uninfected mouse brain tissue. As shown in Figure 3A, whole brain homogenates constitutively expressed robust levels of DAI. Interestingly, both GFAP+ astrocytes and CD11b+ microglia/macrophages isolated from uninfected brain tissue demonstrated only very low DAI expression levels (Figure 3B). This apparent discrepancy suggested that other major CNS cell types, such as neurons, might constitutively express DAI and this hypothesis was supported by the observation that resting CATH.a neuron-like cells expressed high levels of this molecule (Figure 3A).

Furthermore, we assessed glial DAI expression following *in vivo *HSV-1 infection. To confirm that i.n. HSV-1 administration infects glial cells in situ we determined the expression of HSV-1 glycoprotein G1 (gG1) in microglia and astrocytes isolated from infected and sham-infected mice. As shown in Figure 3C, this HSV-1 gene product was absent in cells derived from sham infected mice but was readily detectable in glial cells isolated from the brains of infected animals with astrocytes exhibiting higher levels than microglia/macrophages. Importantly, *in vivo *HSV-1 challenge elicited marked increases in DAI expression in both microglia/macrophages (24.8 fold) and astrocytes (8.5 fold) isolated from the brains of infected animals (Figure 3B) in the absence of significant increases in levels of this viral sensor in total brain protein isolates (Figure 3A). Finally, we assessed the expression of the DAI downstream effector molecules RIP3 and STING in glia isolated from HSV-1 infected and sham infected mice. As shown in Figure 3D, microglia/macrophages and astrocytes isolated from uninfected brain tissue showed detectable levels of RIP3 and STING. Consistent with our *in vitro *findings, *in vivo *HSV-1 infection failed to elicit significant changes in RIP3 expression by either cell type. Interestingly, and in contrast to our studies in cultured cells, STING expression was significantly elevated in both microglia/macrophages and astrocytes following in vivo HSV-1 infection (Figure 3D). While it is likely that STING expression is regulated in vivo by as yet unidentified factors, it must be noted that resting cultured glial cells express high levels of this molecule (Figure 2C), perhaps due to in vitro culture conditions and/or adherence to plastic, and so further increases in STING expression may not be possible in such cells.

### B-DNA induces inflammatory mediator production by primary murine microglia and astrocytes

**Figure 4:**
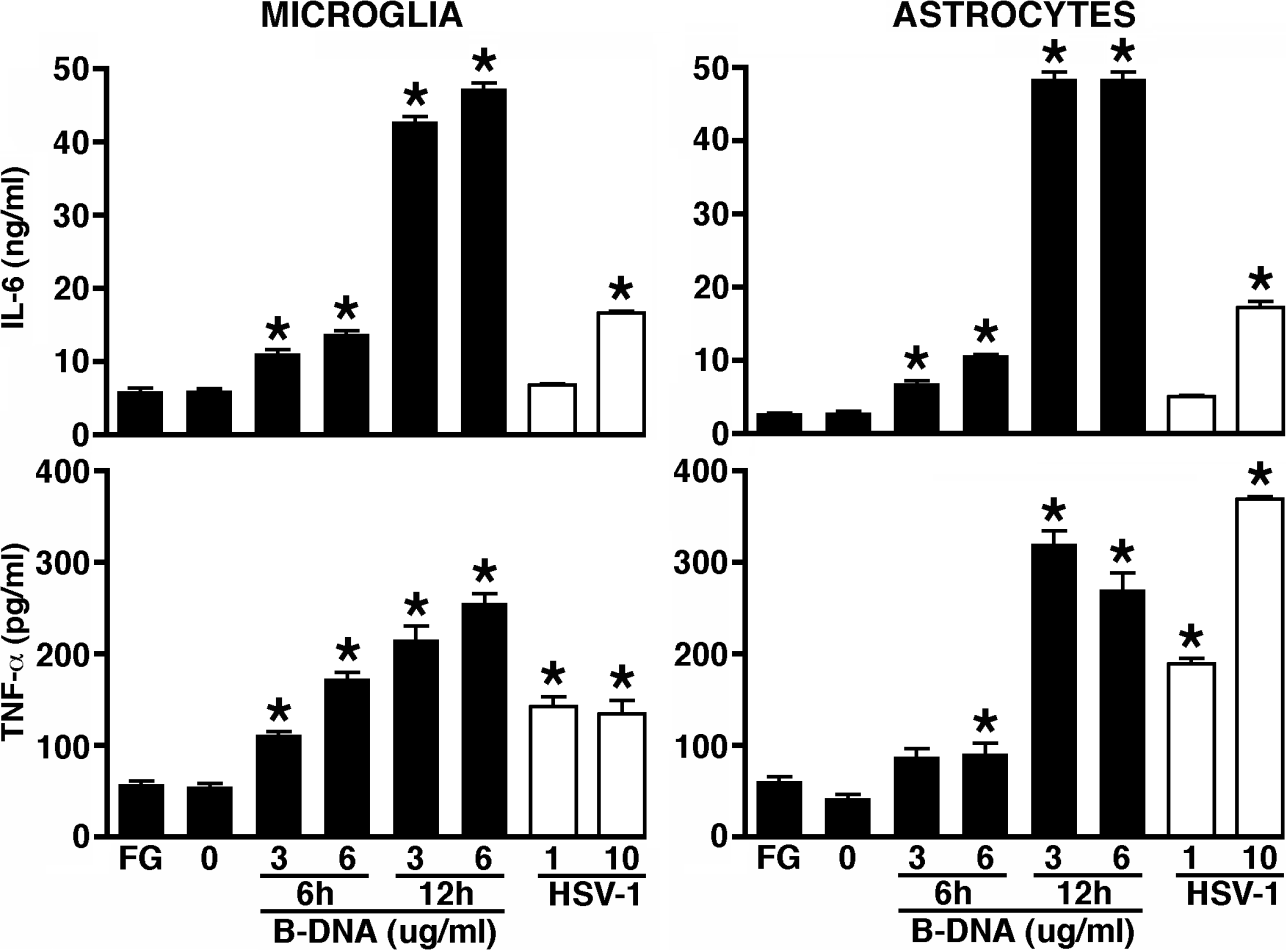
**A specific ligand for DAI induces inflammatory cytokine production by isolated cultures of microglia and astrocytes**. Microglia and astrocytes were untreated (0), treated with transfection reagent alone (FG), or transfected with the DAI ligand B-DNA (3 or 6 ug/ml). At 6 and 12 hours following transfection culture supernatants were collected and IL-6 and TNF-α content was assessed by specific capture ELISA. For comparison purposes inflammatory cytokine production was assessed at 24 hours following HSV-1 infection (MOI of 1 and 10). Data is expressed as mean +/- SEM (n = 6) and an asterisk indicates a statistically significant difference from levels produced by unstimulated cells (p < 0.05).

To begin to determine whether DAI is functional in glial cells, we have assessed the sensitivity of cultured astrocytes and microglia to intracellular administration of poly(dA:dT) double stranded B-DNA. This molecule is a synthetic DNA that displays a helical B-form configuration in solution. Therefore, poly(dA:dT) double stranded B-DNA resembles the form of HSV-1 genomic DNA in infected cells, if not the specific sequence. Importantly, this molecule has previously been demonstrated to strongly elicit innate immune responses in a DAI-dependent manner in other cell types [17,18]. As shown in Figure 4, B-DNA administration induced production of the inflammatory cytokines TNF-a and IL-6 by primary microglia or astrocytes as rapidly as 6 hours post-transfection at levels that matched or exceeded those elicited following 24-hour HSV-1 challenge.

### DAI knockdown attenuates HSV-induced inflammatory cytokine production by primary glia

**Figure 5:**
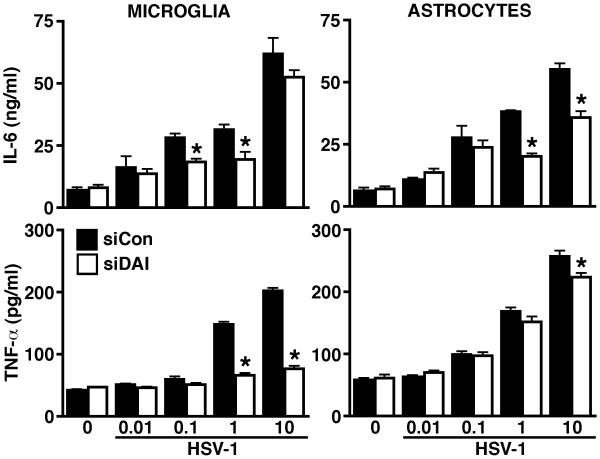
**DAI knockdown attenuates HSV-1-induced inflammatory cytokine production by murine glial cells**. Microglia and astrocytes were untreated (0), or transfected with siRNA targeting DAI (siDAI) or scrambled siRNA (siCon). At 72 hours following transfection, cells were exposed to HSV-1 (MOI of 0.01, 0.1, 1, and 10) and levels of TNF-α and IL-6 in the culture supernatants were assessed at 24 hours post-infection. Data is expressed as mean +/- SEM (n = 3) and an asterisk indicates a statistically significant difference from levels produced by cells treated with scrambled siRNA (p < 0.05).

To confirm the functional status of DAI in glial cells and to begin to determine the relative importance of this innate immune sensor in their responses to neurotropic DNA viruses we have assessed the effect of DAI knockdown on inflammatory cytokine production by HSV-1 challenged microglia and astrocytes. As shown in Figure 5, siRNA directed against DAI significantly attenuated HSV-1 induced TNF-α and IL-6 production by murine microglia. Such an approach also markedly reduced IL-6 production by HSV-1 infected astrocytes but was not as effective in reducing TNF-α production by these cells, where a statistically significant reduction was only observed at the highest viral MOI used (Figure 5).

### DAI is required for virally-induced production of neurotoxic mediators by microglia and astrocytes

**Figure 6:**
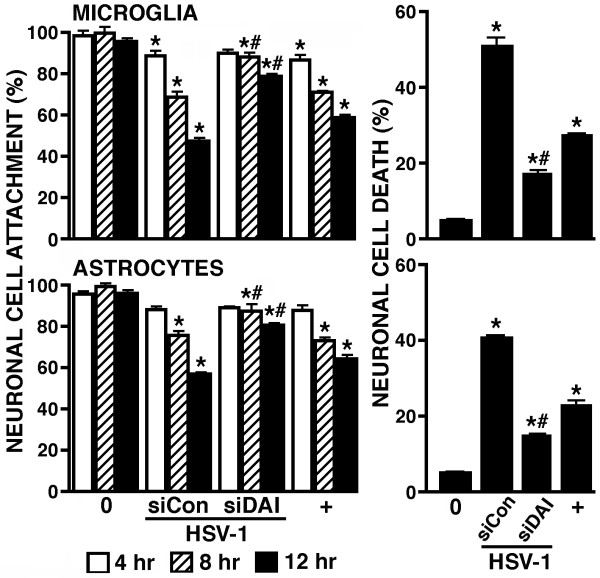
**HSV-1 induces the production of soluble factor(s) by glial cells that elicits neuronal cell damage in a DAI dependent manner**. Primary microglia or astrocytes were untreated (0) or transfected with siRNA targeting DAI (siDAI) or scrambled siRNA (siCon). At 72 hours following the transfection protocol, cells were uninfected or infected with HSV-1 (MOI of 1) and cultured for a further 24 hours. Filtered conditioned media from these cells was placed on CATH.a cells and the number of attached neuronal cells was monitored at 4, 8 and 12 hours post-infection prior to assessment of neuronal cell death by trypan blue exclusion at 12 hours post-infection. For comparison purposes neuronal cell attachment and death was assessed following addition of media spiked with recombinant TNF-α (65 pg/ml: +). Data is expressed as mean +/- SEM (n = 3). Asterisks indicate a statistically significant difference in the number of attached cells or degree of cell death from that seen in unstimulated cells while pound symbols indicate a statistically significant difference in these parameters between cells treated with siRNA directed against DAI and those that received scrambled siRNA (p < 0.05).

To begin to establish a role for DAI in the inflammatory CNS damage associated with neurotropic DNA viral infections, we have assessed the effect of DAI knockdown on the production of soluble neurotoxic mediators by microglia and astrocytes following HSV-1 infection. As shown in Figure 6, HSV-1 induced the production of soluble mediators by microglia that decreased CATH.a neuron-like cell viability as assessed by changes in cell attachment and trypan blue exclusion in an MOI and time dependent manner. Importantly, virally-induced neurotoxic mediator production was significantly attenuated in cells transfected with siRNA targeting DAI (siDAI) as compared to control cells that were transfected with scrambled non-specific siRNA (siCon) (Figure 6). Interestingly, HSV-infected primary astrocytes also produced a substance that elicited neuronal detachment and death, and production of such neurotoxic mediators was similarly attenuated in cells transfected with siRNA targeting DAI (Figure 6). Together, these data point to a key role for DAI in the neurotoxic immune responses of microglia and astrocytes to DNA virus infection and may represent an important component in the inflammation and damage associated with viral encephalitis.

## Discussion

Several members of the herpesvirus family including human herpesvirus 6, HSV-1, and HSV-2 can elicit damaging CNS inflammation [2,32]. Acute HSV -1 infection or the reactivation of latent virus in the trigeminal ganglion can lead to the development of severe encephalitis that is associated with a high degree of morbidity and mortality. While acyclovir is currently employed in the treatment of HSV encephalitis, drug-resistant strains of HSV-1 are beginning to emerge [33,34]. Furthermore, despite improvements in diagnosis such infections are associated with a 30% mortality rate and 62% of survivors recover with severe neurological deficits [33,35,36]. The treatment of HSV-1 associated encephalitis is especially challenging due to the rapid onset of disease and development of irreversible neurological damage in otherwise healthy individuals. These characteristics suggest that the innate immune responses of resident CNS cells play a pivotal role in disease progression, a notion that is supported by the ability of human microglia to produce key inflammatory mediators in response to *in vitro *HSV challenge or following *in vivo *infection [37-40]. However, much of our current understanding of HSV-1 encephalitis pathogenesis comes from rodent models in which intranasal HSV-1 administration results in an acute necrotizing encephalitis that closely resembles human disease [41-45]. In these models, HSV-1 infects both neurons and glial cells and elicits inflammatory mediator production that precedes leukocyte infiltration into the CNS [41,46]. Such findings are consistent with the recognized ability of other viral pathogens to induce inflammatory cytokine production by microglia [47] and the susceptibility and responsiveness of astrocytes to productive HSV-1 infection [8,48]. However, the mechanisms by which resident CNS cells recognize DNA viral pathogens such as HSV-1 have not been fully defined.

We and others have demonstrated that microglia and astrocytes express an array of cell surface and endosomal innate pattern recognition receptors, including TLR2 [9,21], TLR3 [49,50], TLR7 [50], and TLR9 [50], that are capable of recognizing viral motifs [51]. Importantly, each of these cell surface sensors has been implicated in the perception of HSV by a variety of cell types [13,14,51,52]. However, it is becoming increasingly apparent that cells, including glia, possess intracellular sensors that can detect compromise of the cytosolic compartment. We have recently demonstrated that murine and human glial cells functionally express retinoic acid-inducible gene I (RIG-I) and melanoma differentiation-associated antigen 5 (MDA5) [23,24], two members of the RIG-I-like family of helicases that have been shown to function as intracellular pattern recognition receptors for replicative viral RNA motifs [51,53]. It is possible that such receptors may also indirectly serve as sensors for viral and/or bacterial DNA via the actions of RNA polymerase III [54] although a role for this pathway in HSV recognition by immune cells remains controversial [55]. Interestingly, a number of cytosolic proteins including DAI have been described that can directly mediate cellular responses to dsDNA [17,18,53]. It has therefore been suggested that such sensors could play a critical role in the perception of viral DNA and this notion has been supported by the report that DAI mediates immune molecule production by HSV-1-infected murine fibroblasts [17].

In the present study, we provide the first evidence that glial cells express DAI. Resting cultures of primary microglia expressed low levels of this intracellular viral sensor, a finding that is consistent with the very low DAI expression observed in *ex vivo *microglia. In contrast, cultured astrocytes constitutively expressed robust levels of this molecule, although it should be noted that this might be attributable, in part, to our *in vitro *culture conditions, as astrocytes acutely isolated from uninfected mice expressed somewhat lower DAI levels. Importantly, DAI expression was significantly elevated in microglia and astrocytes following either *in vitro *or *in vivo *HSV-1 challenge. Such upregulation was not specific to this neurotropic alphaherpesvirus as the leukotropic gammaherpesvirus, MHV-68, was also capable of elevating DAI expression by both cell types. As such, it is possible that glial perception of DNA viruses via this sensor could promote further DAI expression in a feed-forward manner. The constitutive expression of this viral sensor by glial cells and its upregulation following viral challenge provide circumstantial evidence of a role for DAI in glial responses to DNA viral pathogens. This notion is further supported by the robust constitutive expression by microglia and astrocytes of IPS-1 [23], STING, and RIP3, reportedly critical downstream effector molecules for DAI [29-31]. Importantly, we have confirmed that DAI is functional in primary murine glia by showing that cytoplasmic administration of the DAI ligand, B-DNA, is a potent stimulus for the production of key inflammatory mediators by both microglia and astrocytes. Finally, we have demonstrated a major role for this intracellular viral sensor in the immune responses of primary murine glia to a clinically relevant neurotropic DNA virus by demonstrating that DAI knockdown significantly and specifically inhibits HSV-1-induced inflammatory cytokine production by these cells.

Host responses to viral CNS infections are increasingly recognized to play a major role in disease pathology and the neuroinflammation elicited by HSV-1 has been suggested to underlie the neurological damage associated with infection [3-7]. However, it not clear whether this inflammatory damage is mediated primarily by infiltrating leukocytes or by the responses of the resident cells of the CNS themselves. In support of the latter possibility, activated glial cells are known to be capable of producing toxic mediators that can cause widespread CNS damage [39]. Furthermore, the rapidity with which HSV-1 travels from the initial site of infection to the brain all but assures escape from recognition by adaptive immune cells. Based on these observations, it appears likely that the production of cytotoxic substances by HSV-1 challenged glial cells could play a significant role in neuronal cell dysfunction and/or loss and contribute to the neuropathology associated with HSV-1 encephalitis. In the present study, we have demonstrated that soluble factor(s) released by HSV-infected microglia and astrocytes elicit neuronal cell damage/death and that the production of this factor(s) is dependent, at least in part, on the expression of DAI. While the identification of this/these neurotoxic factor(s) is ongoing in our laboratory, we have shown that TNF-a is released by HSV-1 infected glia (Figure 4) and this cytokine can elicit neuronal cell death (Figure 6). In contrast, we have found that another likely candidate, nitric oxide, is not released by either microglia or astrocytes following HSV-1 infection as assessed by culture medium nitrite content (data not shown) and is therefore unlikely to be a contributing factor to neuronal cell death. Irrespective of the mediator(s) involved, our findings directly implicate DAI in the initiation of inflammatory immune responses by glial cells and suggest a novel mechanism underlying the neuropathology associated with acute DNA viral infections of the CNS.

## Conclusions

**Figure 7:**
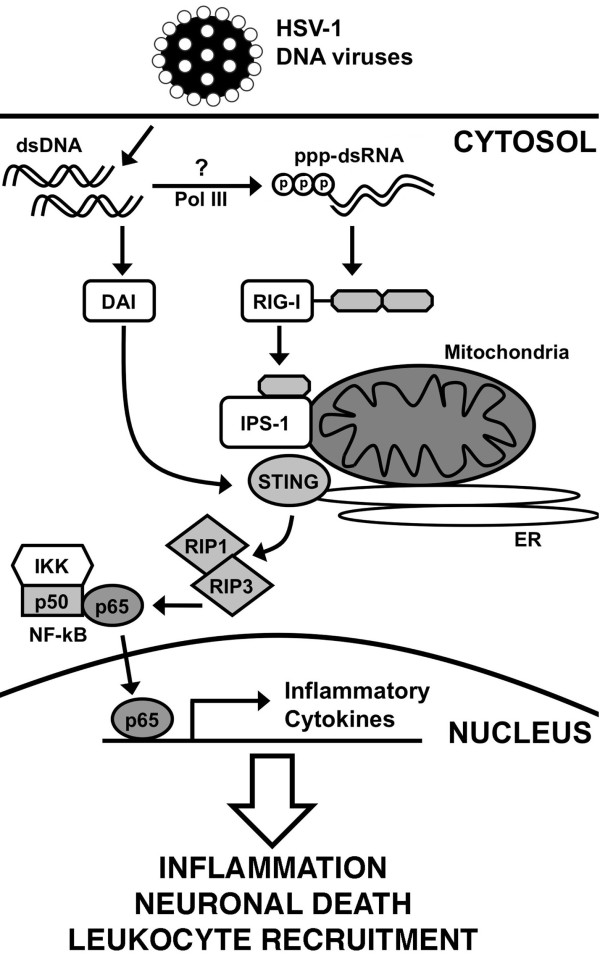
**Proposed model by which glia recognize neurotropic DNA viruses such as HSV-1 and elicit inflammatory CNS damage**. Replicating DNA viruses generate genomic DNA that serves as a ligand for DAI. DAI then associates with IPS-1 and STING, subsequently activating NF-kB via the actions of RIP1 and RIP3. In addition, it is possible that RNA polymerase III (Pol III) in the cytosol could transcribe viral DNA into dsRNA containing 5'-triphosphate (ppp-dsRNA), that could be recognized by RIG-I and similarly activate NF-kB via IPS-1 and STING. Activated NF-kB subunits then translocate to the nucleus and initiate the production of inflammatory mediators. These soluble mediators could then promote inflammation, increase blood-brain-barrier permeability, recruit leukocytes into the CNS, and directly or indirectly initiate neuronal cell damage and/or death.

Based upon our results we propose the model shown in Figure 7. We suggest that neurotropic double-stranded DNA viruses such as HSV-1 infect microglia and astrocytes and replicate within them, generating genomic DNA within the cytoplasm of each cell type. These viral DNA motifs are then recognized by DAI, leading to the activation of downstream effector molecules including IPS-1, STING, and RIP3. Their activation ultimately liberates the RelA subunit of NF-kB allowing it to translocate to the nucleus and to initiate the *de novo *production of soluble inflammatory mediators. In addition, it is possible that RNA polymerase III in the cytosol could transcribe the viral DNA templates into double stranded RNA containing 5'-triphosphate which, in turn, could be recognized by RIG-I and similarly activate NF-kB via the adaptor molecules IPS-1 and STING [54]. However, it should be noted that at least one study suggests that such a pathway does not appear to play a significant role in the responses of some cell types to HSV infection [55]. Once production is initiated, cytokines such as TNF-α and IL-6 would be anticipated to promote inflammation and to increase blood-brain barrier permeability, facilitating leukocyte recruitment into the CNS. In addition, these soluble inflammatory mediators could also initiate neuronal cell loss, either directly or via activation of resident/infiltrating myeloid cells. As such, the functional expression of DAI by glial cells may represent an important innate immune mechanism underlying the rapid and potentially lethal inflammation associated with neurotropic DNA virus infection.

## Competing interests

The authors declare that they have no competing interests.

## Authors' contributions

SRF helped to conceive the study, prepared cell cultures and carried out the *in vitro *and *in vivo *experiments, performed data analysis, and drafted the manuscript. VSC participated in the performance of the *in vivo *viral infections and the isolation of CNS cells. MM participated in the preparation of viral stocks and determination of viral titers. IM helped to conceive the study, contributed to the experimental design, and edited the final manuscript. All authors read and approved the final version of the manuscript.
